# Which patients with advanced respiratory disease die in hospital? A 14-year population-based study of trends and associated factors

**DOI:** 10.1186/s12916-016-0776-2

**Published:** 2017-02-01

**Authors:** Irene J. Higginson, Charles C. Reilly, Sabrina Bajwah, Matthew Maddocks, Massimo Costantini, Wei Gao, on behalf of the GUIDE_Care project

**Affiliations:** 1 Cicely Saunders Institute of Palliative Care, Policy & Rehabilitation, King’s College London, Bessemer Road, London, SE5 9PJ UK; 2 Arcispedale Santa Maria Nuova-IRCCS, Viale Umberto I, 50 – 42123, Reggio Emilia, Italy

**Keywords:** Hospital, Palliative care, End of life care, Chronic obstructive pulmonary disease, Interstitial pulmonary diseases, Interstitial lung disease, Respiratory, Policy, Place of death

## Abstract

**Background:**

Strategies in many countries have sought to improve palliative care and reduce hospital deaths for non-cancer patients, but their effects are not evaluated. We aimed to determine the trends and factors associated with dying in hospital in two common progressive respiratory diseases, and the impact of a national end of life care (EoLC) strategy to reduce deaths in hospital.

**Methods:**

This population-based observational study linked death registration data for people in England dying from chronic obstructive pulmonary disease (COPD) or interstitial pulmonary diseases (IPD). We plotted age- and sex-standardised trends, assessed during the pre-strategy (2001–2004), first strategy phase (2004–2008), and strategy intensification (2009–2014) periods, and identified factors associated with hospital death using multiple adjusted proportion ratios (PRs).

**Results:**

Over 14 years, 380,232 people died from COPD (334,520) or IPD (45,712). Deaths from COPD and IPD increased by 0.9% and 9.2% annually, respectively. Death in hospital was most common (67% COPD, 70% IPD). Dying in hospice was rare (0.9% COPD, 2.9% IPD). After a plateau in 2004–2005, hospital deaths fell (PRs 0.92–0.94). Co-morbidities and deprivation independently increased the chances of dying in hospital, with larger effects in IPD (PRs 1.01–1.55) than COPD (PRs 1.01–1.39) and dose-response gradients. The impact of multimorbidity increased over time; hospital deaths did not fall for people with two or more co-morbidities in COPD, nor one or more in IPD. Living in rural areas (PRs 0.94–0.94) or outside London (PRs, 0.89–0.98) reduced the chances of hospital death. In IPD, increased age reduced the likelihood of hospital death (PR 0.81, ≥ 85 versus ≤ 54 years); divergently, in COPD, being aged 65–74 years was associated with increased hospital deaths (PR 1.13, versus ≤ 54 years). The independent effects of sex and marital status differed for COPD versus IPD (PRs 0.89–1.04); in COPD, hospital death was associated with being married.

**Conclusions:**

The EoLC strategy appeared to have contributed to tangible reductions in hospital deaths, but did not reach people with multimorbidity and this gap widened over time. Integrating palliative care earlier in the disease trajectory especially﻿ in deprived areas and cities, and where multimorbidity is present, should be boosted, taking into account the different demographic factors in COPD and IPD.

**Electronic supplementary material:**

The online version of this article (doi:10.1186/s12916-016-0776-2) contains supplementary material, which is available to authorized users.

## Background

Chronic diseases and multimorbidity are common and increasing. Respiratory diseases are major contributors, especially chronic obstructive pulmonary disease (COPD) and interstitial pulmonary disease (IPD). More than 3 million people worldwide died of COPD in 2012, representing 6% of all deaths that year [1]. Mortality from IPDs is climbing, with current age-standardized mortality ranging from 4 to 10 per 100,000 population (highest in UK and lowest in Sweden) [2]. Both conditions result in a high use of hospital services across all medical areas, especially among people in advanced stages, when the systemic effects of disease lead to dependency [3]; this leads to high healthcare costs. UK population-based data on admissions suggests that, in IPD, the estimated financial burden of hospitalisation in 2010 was £16.2 million per year [4]. Despite this expenditure, there are concerns that care in advanced disease is suboptimal, inadequately co-ordinated, and with patients suffering an average of 14 symptoms, plus psychological and information concerns [5–8]. For most patients with a progressive illness, the hospital is among the least preferred places of death [9].

In the UK, the National Institute of Clinical Excellence (NICE) published Guidance on Supportive and Palliative Care in 2004 [10], and an extra £50 million was allocated to palliative care services. Building from this Guidance, the End of Life Care (EoLC) Programme was developed to improve care in the last year of life, with a specific goal to reach patients in general medical care and with diseases beyond cancer [11]. Roll out was intensified after 2008 within the EoLC Strategy [12]. The Programme and Strategy prioritised home care as an alternative to hospital, promoting initiatives to elicit preferred place of death and boost support from general practitioners. However, whether policies altered care for those dying from major diseases, apart from cancer [11, 13], is unknown. Where people die is a common quality marker. Whilst place of death has been widely studied internationally in cancer [13–15], only one Spanish study has assessed factors associated with place of death from COPD [16], and none for IPD, despite the greater prevalence of respiratory conditions and the imminent global epidemic [3]. Understanding which factors affect place of death is vital for service planning and care improvement, especially given population ageing, rising chronic diseases worldwide and the high costs of hospital admissions [17]. Information to reduce hospitalisations is needed internationally both to meet patient preferences and to ease healthcare costs [18–21].

Therefore, we aimed to compare the trends and factors associated with place of death in people with two common progressive respiratory diseases. We sought to determine whether hospital deaths for individuals dying from COPD or IPD fell after the Strategy was introduced, and after roll out was intensified. To aid future interventions, we also evaluated which factors affected place of death, and whether these were similar across COPD and IPD.

## Methods

### Study design

Population-based observational study (as per STROBE and RECORD [22] guidelines, Additional file 1) as part of our study of Geographical and Temporal Variations in Place of Death in England (GUIDE_Care) [23].

### Data sources

The Office for National Statistics death registrations in England, which detail decedents’ age, sex, marital status, usual residence, place and year of death, and, based on the clinician’s death certificate, the underlying and contributing causes of death using the International Classification of Diseases Tenth Revision (ICD-10), employed since 2001.

Office for National Statistics death registration records were linked with area level indices of multiple deprivation (IMD) 2010 [24]. The IMD 2010 is a composite measure of deprivation, providing a weighted average of seven domains: income, employment, health and disability, education, skills and training, living environment and crime, and barriers to housing and services. These are based on the Lower Super Output Area (LSOA) of the decedents’ usual residence. There are 32,482 LSOAs in England, with each area having a minimum of 1000 residents and an average of 1500. LSOAs were grouped into quintiles based on their IMD scores [13, 24, 25].

### Study population

All deaths between 2001 and 2014 (inclusive) with COPD or IPD as an underlying cause of death (ICD-10 codes: J40-J44, J47 (COPD); J84 (Interstitial pulmonary disease, encompassing all progressive fibrotic interstitial lung diseases including idiopathic pulmonary fibrosis and idiopathic interstitial pneumonia)) were extracted.

### Variables

The main outcome was place of death grouped into six categories: hospital, home, hospice (an inpatient specialist palliative care unit, freestanding or clearly specified within a hospital, 75% are voluntary, 25% NHS managed), nursing home, care home or residential home, and elsewhere [13].

Explanatory variables were age at death (≤54, 55–64, 65–74, 75–84, 85+), sex (men, women), year of death (grouped into 2001–2004 (pre-Strategy), 2005–2008 (Strategy first phase, which included the implementation of the NICE Guidance and first phase of the EoLC Programme and Strategy), and 2009–2014 (Strategy intensification)), marital status (married, widowed, divorced, single, not stated/unknown), number of co-morbidities assessed from contributory cause(s) of death (0, 1, 2, 3, 4+), type of settlement (rural, urban), socioeconomic status (as measured by IMD of area of residence), and region (defined by Clinical Senate, 2013). We analysed age as an ordered five-category rather than a continuous variable to aid interpretation and comparison with other studies; category boundaries were chosen based on the data distribution [26–28].

### Statistical analysis

We plotted the time trend of age- and sex-standardised proportion of deaths in hospital for COPD and IPD. Proportions were standardised using the 2010–2015 mortality structure for more developed countries from the United Nations standard population [29].

We used Modified Poisson Regression to evaluate the relationship between place of death and potential explanatory variables (selected from those available, according to existing literature and univariable analysis results), including age, sex, marital status, co-morbidity, year of death, IMD, rural/urban indicator and region of the usual residential address (Table 2). The dependent variable was binary (1 = hospital, 0 = non-hospital). We focused on hospital death as this was most common and reducing it was a target of the Strategy. The strength of association was measured using proportion ratios (PRs). Two separate models were constructed for COPD and IPD. The Modified Poisson regression was chosen over the binomial model as the latter failed to converge in IPD [30]. Sensitivity analysis entered year of death as a continuous rather than categorical variable.

All analyses were performed using the SAS 9.4 (SAS Institute, Cary, NC, USA).

## Results

Over the 14 years, 334,520 people died from COPD and 45,712 from IPD (Table 1), representing 5.3% and 0.7% of the total 6,368,760 non-accidental deaths during the period. Annual deaths from COPD increased slightly from 23,303 (2001–2004) to 24,717 (2009–2014), representing a yearly increase of 0.9%. The annual number of IPD deaths, although much smaller, almost doubled from 2403 (2001–2004) to 4091 (2009–2014); representing a yearly increase of 9.2%. Across both conditions, more than 65% of deaths occurred among people aged over 75 years.

**Table 1:** Sociodemographic and clinical characteristics (count, %) of patients who died from chronic obstructive pulmonary disease (COPD) and interstitial pulmonary disease (IPD), England 2001–2014

Variable	Value	COPD *N* = 334,520	IPD *N* = 45,712
Age in years	Mean (SD)	78.2 (9.7)	77.0 (10.5)
	Min–max	0–108	0–105
Age group	0–54	5593 (1.7)	1284 (2.8)
	55–64	25,283 (7.6)	3503 (7.7)
	65–74	73,095 (21.9)	11,180 (24.5)
	75–84	139,030 (41.6)	18,893 (41.3)
	85+	91,519 (27.4)	10,852 (23.7)
Sex	Male	175,226 (52.4)	28,231 (61.8)
	Female	159,294 (47.6)	17,481 (38.2)
Marital status	Married	123,808 (37.0)	24,342 (53.3)
	Widowed	146,706 (43.9)	15,280 (33.4)
	Divorced	36,137 (10.8)	3129 (6.8)
	Single	25,240 (7.5)	2651 (5.8)
	Not stated/unknown	2629 (0.8)	310 (0.7)
Number of contributing causes of death (co-morbidities)	0	42,769 (12.8)	9547 (20.9)
	1	117,532 (35.1)	15,718 (34.4)
	2	94,070 (28.1)	11,166 (24.4)
	3	47,641 (14.2)	5496 (12.0)
	4+	32,508 (9.7)	3785 (8.3)
Settlement	Urban	281,013 (84.0)	36,938 (80.8)
	Rural	53,507 (16.0)	8774 (19.2)
Indices of Multiple Deprivation	1 – Most deprived	93,671 (28.0)	8632 (18.9)
	2	76,635 (22.9)	8769 (19.2)
	3	65,336 (19.5)	9698 (21.2)
	4	55,901 (16.7)	9783 (21.4)
	5 – Least deprived	42,969 (12.8)	8829 (19.3)
Clinical senate (i.e. region)	Cheshire & Merseyside	20,004 (6.0)	2707 (5.9)
	East Midlands	28,848 (8.6)	4325 (9.5)
	East of England	33,243 (9.9)	4693 (10.3)
	Greater Manchester, Lancashire and south Cumbria	33,367 (10.0)	4273 (9.3)
	London	36,184 (10.8)	4091 (8.9)
	North East, north Cumbria, and the Hambleton & Richmondshire districts of North Yorkshire	26,546 (7.9)	3185 (7.0)
	South East Coast	27,489 (8.2)	3803 (8.3)
	South West	28,042 (8.4)	4270 (9.3)
	Thames Valley	9265 (2.8)	1378 (3.0)
	Wessex	15,930 (4.8)	2571 (5.6)
	West Midlands	36,088 (10.8)	5473 (12.0)
	Yorkshire & The Humber	39,514 (11.8)	4943 (10.8)
Year of death (average/year)	2001–2004	93,212 (23,303/year)	9612 (2403/year)
	2005–2008	93,008 (23,252/year)	11,557 (2889/year)
	2009–2014	148,300 (24,717/year)	24,543 (4091/year)
Place of death	Hospital	225,024 (67.3)	32,066 (70.1)
	Home	66,510 (19.9)	8748 (19.1)
	Hospice	2882 (0.9)	1330 (2.9)
	Nursing home	21,142 (6.3)	1935 (4.2)
	Care/Residential home	16,358 (4.9)	1318 (2.9)
	Elsewhere	2604 (0.8)	315 (0.7)

Comparison of IPD versus COPD all *P* < 0.0001

Hospital was the most common place of death (67.3% for COPD, 70.1% IPD), followed by home (19.9% COPD, 19.1% IPD). Deaths within hospices accounted for just 0.9% of COPD and 2.9% of IPD cases (Table 1).

**Fig. 1:**
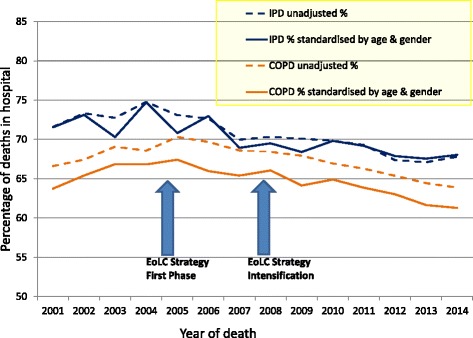
Time trends of percentage of deaths in hospital by year for chronic obstructive pulmonary disease (COPD) and interstitial pulmonary disease (IPD). Unadjusted and standardised by age and sex against the United Nations mortality standard population

The pattern of higher proportions of hospital deaths in IPD was consistent over the years, even when standardised by age and sex. For both groups, the proportion of hospital deaths peaked between 2003 and 2005 (Fig. 1). The proportion of age- and sex-standardised deaths in hospital fell slightly between 2005 and 2014 for people with COPD (from 67% to 61%) and IPD (from 71 to 68%).

In multivariable analysis (Table 2), hospital deaths reduced significantly over time in both COPD and IPD, with a fall occurring after 2005. Female sex was independently associated with higher hospital deaths in COPD (PR 1.04) but lower in IPD (PR 0.97). In COPD, there was a U-shaped relationship regarding age, with those aged 65–74 years having the highest hospital deaths (PR 1.12); whereas in IPD, increased age was independently associated with fewer hospital deaths (PR 0.81). Being married increased the chance of dying in hospital for COPD but not for IPD (Table 2).

**Table 2:** Factors associated with place of death^a^ (hospital versus non-hospital) in patients who died from chronic obstructive pulmonary disease ((COPD) and interstitial pulmonary disease (IPD), England 2001–2014

Variable (Ref)	Value	Underlying cause of death			
		COPD		IPD	
		Unadjusted PR (95% CI)	Adjusted PR	Unadjusted PR	Adjusted PR
Age (≤54)	55–64	1.07 (1.05–1.10)	1.07 (1.04–1.09)	0.95 (0.92–0.98)	0.95 (0.92–0.98)
	65–74	1.13 (1.11–1.16)	1.12 (1.10–1.14)	0.89 (0.87–0.92)	0.90 (0.88–0.93)
	75–84	1.13 (1.11–1.16)	1.11 (1.08–1.13)	0.85 (0.83–0.87)	0.85 (0.83–0.88)
	85+	1.04 (1.02–1.06)	1.02 (1.00–1.04)	0.82 (0.79–0.84)	0.81 (0.79–0.84)
Sex (Male)	Female	1.02 (1.01–1.02)	1.04 (1.03–1.04)	0.96 (0.95–0.97)	0.97 (0.95–0.98)
Marital status (Married)	Divorced	0.93 (0.92–0.93)	0.92 (0.91–0.93)	1.02 (0.99–1.04)	0.99 (0.96–1.01)
	Single	0.89 (0.88–0.90)	0.89 (0.88–0.90)	1.02 (1.00–1.05)	0.98 (0.96–1.00)
	Widowed	0.95 (0.94–0.95)	0.95 (0.94–0.95)	0.98 (0.97–0.99)	1.01 (0.99–1.02)
	NS/unknown	0.84 (0.82–0.87)	0.83 (0.81–0.86)	0.96 (0.88–1.03)	0.87 (0.80–0.94)
Year of death (2001–2004)	2005-2008	1.02 (1.01–1.03)	1.01 (1.00–1.02)	0.98 (0.96–0.99)	0.97 (0.95–0.99)
	2009-2014	0.97 (0.96–0.97)	0.94 (0.94–0.95)	0.93 (0.92–0.95)	0.92 (0.90–0.93)
No. contributing causes of death (co-morbidities) (0)	1	1.04 (1.03–1.05)	1.06 (1.05–1.07)	1.26 (1.23–1.28)	1.26 (1.24–1.29)
	2	1.08 (1.07–1.09)	1.10 (1.09–1.11)	1.32 (1.30–1.35)	1.33 (1.31–1.36)
	3	1.20 (1.19–1.21)	1.23 (1.22–1.24)	1.41 (1.38–1.44)	1.42 (1.39–1.46)
	4+	1.35 (1.34–1.36)	1.39 (1.37–1.40)	1.52 (1.49–1.56)	1.55 (1.52–1.59)
Indices of Multiple Deprivation 5 (Least deprived)	1 (Most deprived)	1.06 (1.06–1.07)	1.05 (1.04–1.05)	1.15 (1.13–1.17)	1.08 (1.05–1.10)
	2	1.05 (1.04–1.06)	1.04 (1.03–1.05)	1.10 (1.08–1.12)	1.05 (1.03–1.08)
	3	1.03 (1.02–1.04)	1.03 (1.02–1.04)	1.05 (1.03–1.08)	1.03 (1.01–1.05)
	4	1.01 (1.00–1.02)	1.01 (1.00–1.02)	1.02 (1.00–1.04)	1.01 (0.99–1.03)
Rural/urban (Urban)	Rural	0.93 (0.92–0.94)	0.95 (0.94–0.96)	0.90 (0.89–0.92)	0.94 (0.93–0.96)
Region (London)	Cheshire & Merseyside	0.97 (0.96–0.98)	0.96 (0.95–0.97)	0.92 (0.89–0.94)	0.92 (0.90–0.95)
	East Midlands	0.95 (0.94–0.96)	0.95 (0.94–0.96)	0.91 (0.88–0.93)	0.94 (0.91–0.96)
	East of England	0.96 (0.95–0.97)	0.96 (0.96–0.97)	0.90 (0.87–0.92)	0.93 (0.91–0.96)
	Greater Manchester, Lancashire and south Cumbria	0.98 (0.97–0.99)	0.97 (0.97–0.98)	0.97 (0.95–0.99)	0.98 (0.96–1.01)
	North East, north Cumbria, and the Hambleton & Richmondshire districts of North Yorkshire	0.96 (0.95–0.97)	0.94 (0.93–0.95)	0.95 (0.92–0.97)	0.96 (0.94–0.99)
	South East Coast	0.92 (0.91–0.93)	0.94 (0.93–0.95)	0.85 (0.82–0.87)	0.89 (0.86–0.91)
	South West	0.89 (0.88–0.90)	0.91 (0.90–0.92)	0.84 (0.82–0.87)	0.89 (0.86–0.91)
	Thames Valley	0.94 (0.92–0.95)	0.96 (0.94–0.98)	0.87 (0.84–0.91)	0.93 (0.89–0.97)
	Wessex	0.93 (0.92–0.95)	0.94 (0.93–0.95)	0.90 (0.88–0.93)	0.95 (0.92–0.98)
	West Midlands	0.98 (0.97–0.99)	0.96 (0.95–0.97)	0.94 (0.92–0.97)	0.95 (0.93–0.98)
	Yorkshire & The Humber	0.93 (0.92–0.94)	0.92 (0.91–0.93)	0.91 (0.89–0.94)	0.93 (0.91–0.95)

^a^The association is measured by proportion ratios (PRs) and 95% confidence intervals. PR > 1 indicates a higher probability of hospital death, < 1 lower chance of hospital death, PR = 1 indicates no association. The adjusted PRs were derived from modified Poisson regression model, adjusting for the listed variables

**Fig. 2:**
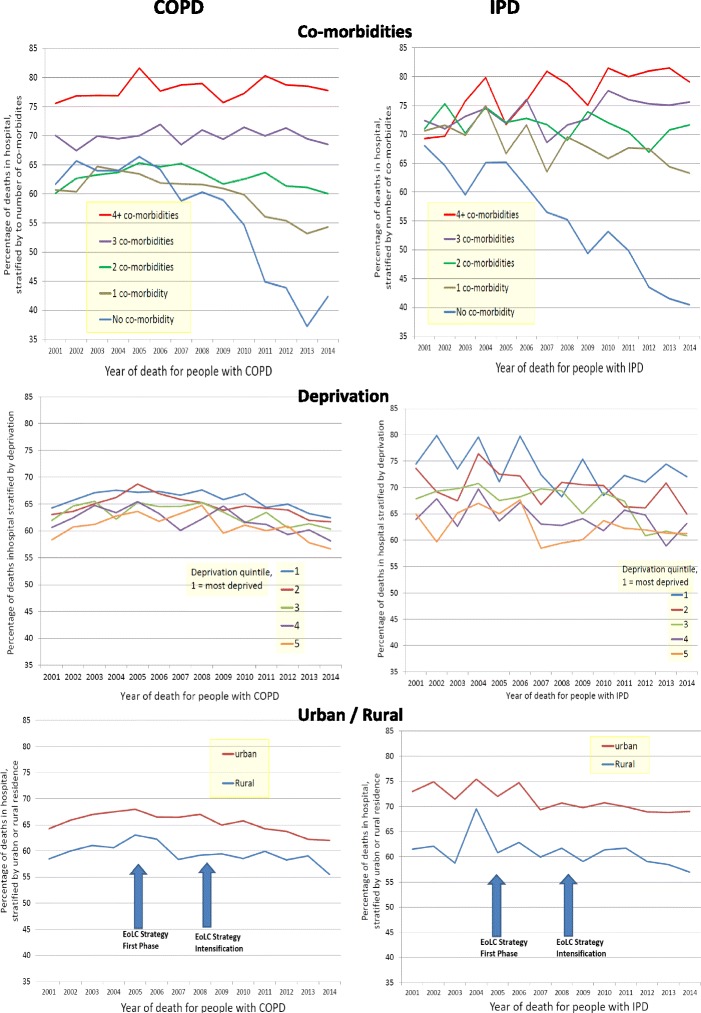
Time trends of percentage of deaths in hospital for COPD and IPD stratified according to number of co-morbidities, deprivation score and living in urban or rural areas. Percentages are age and gender standardised against the United Nations mortality standard population

Having co-morbidities and living in deprived areas independently increased the chance of dying in hospital, with larger effects for IPD (PRs 1.01–1.55) than COPD (PRs 1.01–1.39) and a “dose–response” relationship (Fig. 2). Living in rural areas as opposed to cities reduced the chances of hospital death (PRs 0.94–0.95). When plotted over the period (Fig. 2), after 2005 hospital deaths fell chiefly for people with no co-morbidities. Hospital deaths did not fall for people with two or more co-morbidities in COPD, or with any co-morbidity in IPD. For deprivation and urban areas, the dose–response relationship appeared to stay constant over time (Fig. 2).

Significant variations by region were observed for both COPD and IPD. For COPD and IPD groups, “London” had the highest hospital deaths, and the “South West” and “South East Coast” regions had lower hospital deaths than most other regions. Sensitivity analysis, with year of death as a continuous variable, produced similar results (Table 3).

**Table 3:** Sensitivity analysis. Factors associated with place of death^a^ (hospital versus non-hospital) in patients who died from chronic obstructive pulmonary disease (COPD) and interstitial pulmonary diseases (IPD), England 2001–2014, using year of death as a continuous variable

Variable (Ref)	Value	Underlying Cause of Death			
		COPD		IPD	
		Unadjusted PR (95% CI)	Adjusted PR	Unadjusted PR	Adjusted PR
Age (≤54)	55–64	1.07 (1.05–1.10)	1.07 (1.05–1.09)	0.95 (0.92–0.98)	0.95 (0.92–0.98)
	65–74	1.13 (1.11–1.16)	1.12 (1.10–1.14)	0.89 (0.87–0.92)	0.90 (0.88–0.93)
	75–84	1.13 (1.11–1.16)	1.11 (1.09–1.13)	0.85 (0.83–0.87)	0.85 (0.83–0.88)
	85+	1.04 (1.02–1.06)	1.02 (1.00–1.04)	0.82 (0.79–0.84)	0.82 (0.79–0.84)
Sex (Male)	Female	1.02 (1.01–1.02)	1.04 (1.03–1.05)	0.96 (0.95–0.97)	0.97 (0.95–0.98)
Marital status (Married)	Divorced	0.93 (0.92–0.93)	0.92 (0.92–0.93)	1.02 (0.99–1.04)	0.99 (0.96–1.01)
	Single	0.89 (0.88–0.90)	0.89 (0.88–0.90)	1.02 (1.00–1.05)	0.98 (0.96–1.01)
	Widowed	0.95 (0.94–0.95)	0.95 (0.94–0.95)	0.98 (0.97–0.99)	1.01 (0.99–1.02)
	NS/unknown	0.84 (0.82–0.87)	0.83 (0.81–0.86)	0.96 (0.88–1.03)	0.87 (0.80–0.94)
Year of death	–	0.995 (0.995–0.996)	0.992 (0.992–0.993)	0.993 (0.991–0.994)	0.991 (0.989–0.992)
No. contributing Causes of Death (co-morbidities) (0)	1	1.04 (1.03–1.05)	1.06 (1.05–1.07)	1.26 (1.23–1.28)	1.26 (1.23–1.29)
	2	1.08 (1.07–1.09)	1.10 (1.09–1.11)	1.32 (1.30–1.35)	1.33 (1.31–1.36)
	3	1.20 (1.19–1.21)	1.23 (1.22–1.24)	1.41 (1.38–1.44)	1.42 (1.39–1.46)
	4+	1.35 (1.34–1.36)	1.39 (1.38–1.40)	1.52 (1.49–1.56)	1.55 (1.52–1.59)
Indices of Multiple Deprivation 5 (Least deprived)	1 (Most deprived)	1.06 (1.05–1.07)	1.05 (1.04–1.06)	1.15 (1.13–1.17)	1.08 (1.05–1.10)
	2	1.05 (1.04–1.06)	1.04 (1.03–1.05)	1.10 (1.08–1.12)	1.05 (1.03–1.08)
	3	1.03 (1.02–1.04)	1.03 (1.02–1.04)	1.05 (1.03–1.08)	1.03 (1.01–1.05)
	4	1.01 (1.00–1.02)	1.01 (1.00–1.02)	1.02 (1.00–1.04)	1.01 (0.99–1.03)
Rural/urban (Urban)	Rural	0.93 (0.92–0.94)	0.95 (0.94–0.96)	0.90 (0.89–0.92)	0.94 (0.93–0.96)
Region (London)	Cheshire & Merseyside	0.97 (0.96–0.98)	0.96 (0.95–0.97)	0.92 (0.89–0.94)	0.92 (0.90–0.95)
	East Midlands	0.95 (0.94–0.96)	0.95 (0.94–0.96)	0.91 (0.88–0.93)	0.94 (0.91–0.96)
	East of England	0.96 (0.95–0.97)	0.97 (0.96–0.97)	0.90 (0.87–0.92)	0.93 (0.91–0.96)
	Greater Manchester, Lancashire and south Cumbria	0.98 (0.97–0.99)	0.97 (0.97–0.98)	0.97 (0.95–0.99)	0.98 (0.96–1.01)
	North East, north Cumbria, and the Hambleton & Richmondshire districts of North Yorkshire	0.96 (0.95–0.97)	0.94 (0.93–0.95)	0.95 (0.92–0.97)	0.96 (0.94–0.99)
	South East Coast	0.92 (0.91–0.93)	0.94 (0.93–0.95)	0.85 (0.82–0.87)	0.89 (0.86–0.91)
	South West	0.89 (0.88–0.90)	0.91 (0.90–0.92)	0.84 (0.82–0.87)	0.89 (0.86–0.91)
	Thames Valley	0.94 (0.92–0.95)	0.96 (0.94–0.98)	0.87 (0.84–0.91)	0.93 (0.89–0.97)
	Wessex	0.93 (0.92–0.95)	0.94 (0.93–0.95)	0.90 (0.88–0.93)	0.95 (0.92–0.98)
	West Midlands	0.98 (0.97–0.99)	0.96 (0.95–0.97)	0.94 (0.92–0.97)	0.95 (0.93–0.98)
	Yorkshire & The Humber	0.93 (0.92–0.94)	0.92 (0.91–0.93)	0.91 (0.89–0.94)	0.93 (0.91–0.95)

^a^The association is measured by proportion ratios(PRs) and 95% confidence intervals. PR > 1 indicates a higher probability of hospital death, < 1 lower chance of hospital death, PR = 1 indicates no association. The adjusted PRs were derived from modified Poisson regression model, adjusting for the listed variables

## Discussion

In this large population-based study assessing place of death in respiratory disease over 14 years, we found hospital was the most common place of death, and remained constantly higher for people with IPD (67.5–74.7%, standardised by age and sex) than COPD (61.3–67.4%). During the period following the introduction of the NICE Guidance on Supportive and Palliative Care, and the introduction and intensification of the EoLC Strategy, hospital deaths fell slightly, by 6% for people with COPD and 3% for IPD. In regression analysis, the change after 2005 was significant for both COPD and IPD.

Our design is observational; therefore, as in other population studies, we cannot infer causality. However, our findings meet many of the Bradford-Hill and related criteria [31] for providing evidence supporting a causal relationship. There is strength, consistency, specificity and coherence in our findings. We observed a similar but more pronounced and immediate effect in adults who died from cancer – the traditionally best served condition by palliative and end of life care [13]. Conversely, in children and young people with cancer, a patient population rarely accessing palliative and end of life care services, the Strategy made little impact on where people die [32]. There is strong evidence of temporality, with the changes emerging after the Strategy was introduced and increased after it was intensified.

For both conditions, multimorbidity, social deprivation and living in urban areas were associated with dying in hospital, with larger effects for IPD than COPD. We found a “dose–response” effect, with higher deprivation and multimorbidity producing the largest effects on dying in hospital. Increased age and being female were associated with higher hospital deaths in COPD but lower in IPD. In both groups, being single, widowed or divorced, and living in rural or outside London areas were associated with reduced chances of dying in hospital. These data are ecological, and may be subject to the ecological fallacy, whereby the risk-associations apparent for social deprivation, multimorbidity, or other factors may not accurately reflect the true association between individuals within those groups. However, our findings meet the Bradford-Hill criteria of a dose–response relationship. These associations could be examined further in prospective research.

Our findings suggest the EoLC Strategy contributed to tangible impact in reducing hospital deaths for people with respiratory diseases. However, the effects were mainly for people with few or no co-morbidities; people with two or more co-morbidities had no reduction in hospital deaths over the period. Most healthcare systems are dominantly established for people with individual diseases [33, 34]. Many different specialists can become involved during multimorbidity. This can be duplicative, burdensome and unsafe for patients because of poor coordination and integration, with some patients inversely receiving less care [33, 35, 36]. Another reason might be that the more medical specialists are involved, the higher the chance of the family doctor or general practitioner (GP) having a minor role in the care of the patient, thus being less often aware of the progressive stage of the diseases. Supporting patients with multimorbidity needs knowledge and expertise that a GP may not always have, which may be a reason for why GPs may refer to hospital during crises. These factors could increase hospital admissions and length of stay.

Multimorbidity is increasing globally [37, 38]. In the UK, the number of people with three or more long-term conditions is predicted to rise from 1.9 million in 2008 to 2.9 million in 2018, requiring a major increase in healthcare expenditure [39]. It was particularly concerning that, in our data, there was no fall in hospital deaths for people with multimorbidity, and the disparity widened over time. Therefore, it is essential that future strategies for end of life and palliative care directly address the issue of multimorbidity, and this may require different approaches.

Median survival for people with IPD is poor, at between 2 and 5 years from diagnosis [40]. Prognosis in COPD is better, though around one third die within 3 years following diagnosis [41]. However, it is difficult to prognosticate, as many people have frequent exacerbations of their disease and encounter life-threatening events before death occurs. Prognostication, especially during the last year of life, is problematic when patients have multimorbidity. Patients with COPD and IPD often suffer refractory breathlessness, which can result in panic and distress [42]. Breathlessness is often unpredictable and episodic [5, 43], with multiple other symptoms that can result in accident and emergency attendance [44]. Managing refractory breathlessness and issues in multimorbidity is therefore more complex and time consuming than for single conditions, especially responding to the needs for ‘joined-up’ co-ordination, communication and symptom management [35, 36]. Organising some specific treatments, such as non-invasive ventilation in COPD, also may take time in the community.

Taken together, these findings suggest that earlier palliative care is needed, with an integrated short-term assessment and review. Two challenges exist regarding access to end of life or palliative care for a patient with organ failure. Firstly, when and how to trigger care? As prognostication is difficult, professionals can have ‘prognostic paralysis’, and may postpone discussions with patients about the future. Secondly, patients may not realise or acknowledge that they have a life-limiting disease. This is often not clearly communicated by the healthcare professionals, which implies that preferred place of death is not discussed [45]. Early palliative care could be triggered by multimorbidity and complexity in terms of symptoms and needs rather than waiting until the end of life is apparent or acknowledged. Evidence supports such services; strong evidence supports early integration in cancer [46, 47] and evidence is emerging in respiratory and mixed conditions for integrated breathlessness support services [48, 49], hospital to home [50] and multiprofessional teams [51]. Perhaps multimorbidity should be a specific focus for palliative care. A recent study found the cost-savings of palliative care were largest among patients with multimorbidity, costs were 22% lower than standard care for patients with a co-morbidity score of 2–3 and with 32% lower for those with a score of 4 or higher [52]. There may be a role for tools to understand and elicit discussions earlier in care, including in Intensive Care Units [53, 54], and for palliative care units in the acute hospital where non-invasive ventilation can be provided. Exactly how integration should occur needs to be tailored to the characteristics of the healthcare system and the local resources but evidence suggests that identification based on clinical characteristics is more reliable than relying on clinicians to remember to make referrals [46]. The factors identified in this study, along with the symptom of breathlessness, could be applied to trigger more integrated palliative support, with models such as a breathlessness support service [49]. Hospices, which in the UK provide inpatient specialist palliative care, remained a rare place of death, yet these may be appropriate places outside of hospitals to care for more complex patients with multimorbidity.

Our findings support other research that found deprivation is associated with higher hospital and fewer home deaths in cancer and COPD [15, 55, 56], and studies in COPD suggesting that deprivation and co-morbidity are associated with hospital admission and readmission for acute exacerbation [17, 57, 58]. We could find no literature on deprivation in IPD; ours appears to be the first study to consider this group. It would seem plausible that more hospital admissions may lead to greater chances of dying in hospital. McAllister et al.’s [55] study in Scotland found that winter and socioeconomic deprivation-related factors appear to act synergistically, increasing the rate of COPD admissions to hospital more among deprived people and in winter. The results suggest there may be a role for targeting initiatives in deprived areas and in winter. Interestingly, we found both diseases were still increasing in frequency as a cause of death over the period and for COPD this appears to run contrary to other European trends [59].

Factors such as being widowed or divorced making hospital deaths less likely in COPD are surprising; this is independent of age and may suggest the presence of family members increased the chance of patients being admitted to hospitals. Qualitative work has identified gaps in information and support for patients, families and professionals, who are often invisible to services [60]. The findings highlight more work is needed to support patients and family members at home, who often struggle in knowing what to do when breathlessness escalates [61].

For COPD, our findings of age and sex run contrary to those of a study of 4983 decedents in Andalusia, Spain [16], where older age and female sex were associated with home death. However, the Andalusia study controlled for a smaller number of potential confounders, e.g. deprivation and co-morbidities were not assessed, whereas both were important in our study. It also considered only one year, 2009, and focussed on factors associated with deaths at home versus elsewhere rather than with hospital versus elsewhere. In accordance with our results, the Andalusia study found rural residents were more likely to die at home.

Our study was limited by the nature of data available. We do not have information to address the appropriateness of the place of end of life care and the quality of the care provided. COPD and IPD can be characterised by a trajectory of a prolonged phase of recurrent exacerbations with recovery. Patients and their families are often acutely distressed during an exacerbation. Standard medical treatments are often appropriate in correcting reversible processes, and also in providing symptom relief in these circumstances. Thus, palliative and end of life care strategies may well need to be different for these patients than for cancer patients. Approximately 70% of the patients died in hospital, with patients admitted to hospital rather than remaining at home. Primary care and hospital teams may have thought that admission was the best option [61]. Many services have now moved on from the limitations of applying the generic EoLC strategy to COPD and IPD patients, to a model of care whereby standard medical and palliative care are delivered in parallel, in an integrated way, particularly in diseases where the patient has a good prospect of recovery from an acute exacerbation.

Other research indicates that people with COPD and IPD often miss out on the best care in advanced stages of illness, in the community, in hospital, and from palliative care [8, 62, 63]. Our finding of high hospital use may be a result of lower quality care and forward planning, driving emergency admissions [61, 63, 64]. There is little research on the preferences for place of care and death of people with respiratory disease, but there is no data to indicate that their preferences are especially different from other groups [14]. It is possible that COPD and IPD were misclassified as a cause of death, but as we focussed on recent years, this effect is likely to be minimised. The increase in mortality from IPD has been partly linked to better identification of the disease [2, 4]. The apparent growth in IPD in our study may also be as a result of this trend. It is also possible that classification of IPD and COPD as a cause of death is influenced by setting, and other conditions, such as pneumonia, are more commonly recorded in community settings, thus underestimating COPD and IPD deaths in these settings. However, these limitations would be unlikely to affect the trends over time or the associated factors. The only concern would be if the Strategy helped to identify people with COPD and IPD earlier, which lead to increased community identification and recording as a cause of death. If this occurred, the effect of the Strategy found here would be over-estimated. Multimorbidity is usually defined as the presence of two or more chronic diseases within an individual. We used the reported contributing causes of death to determine the number of co-morbidities. It may have been that only more major co-morbidities were recorded and therefore the number of co-morbidities may be underestimated. However, the presence of the clear trend shows the need to focus more clearly in the future on multimorbidity and its potential role. Our factors and trends point the way to potential interventions to improve care.

## Conclusions

Hospital deaths from COPD and IPD fell by 3–6% in the 8 years following the introduction of the EoLC strategy; however, those with multimorbidity did not show a fall in hospital deaths. Multimorbidity, deprivation, living in cities, and living in London play a greater role in affecting where people with IPD die than those with COPD. Age and sex affect the chance of hospital death differently for COPD and IPD. Being married rather than single, widowed or divorced made hospital death more likely in COPD, but not in IPD. Thus, the results suggest that the EoLC Strategy may have helped to shift some deaths out of hospital for people with respiratory disease but more integrated approaches of earlier palliative care are needed, targeting those at highest risk, especially with multimorbidity, and in deprived areas and cities. Further initiatives and trials are needed to understand and to improve the quality of care for people both in hospital (where most people are dying) and at home.

## Additional file

Additional file 1: Inclusion of information in paper according to RECORD and STROBE statements that should be reported in observational studies using routinely collected health data. (DOCX 21 kb)

## Abbreviations

COPD: chronic obstructive pulmonary disease
EoLC: end of life care
GP: general practitioner
GUIDE_Care: Geographical and Temporal Variations in Place of Death in England (1984-2010) Understanding Trends and Associated Factors to Improve End-of-life Care
ICD-10: International Classification of Diseases Tenth Revision
IMD: Index of Multiple Deprivation
IPD: interstitial pulmonary diseases
LSOA: lower super output area
PR: proportion ratios
RECORD: REporting of studies Conducted using Observational Routinely-collected Data
STROBE: STrengthening the Reporting of Observational studies in Epidemiology
